# Use of Deep Neural Networks to Predict Obesity With Short Audio Recordings: Development and Usability Study

**DOI:** 10.2196/54885

**Published:** 2024-07-25

**Authors:** Jingyi Huang, Peiqi Guo, Sheng Zhang, Mengmeng Ji, Ruopeng An

**Affiliations:** 1 School of Economics and Management Shanghai University of Sport Shanghai China; 2 Brown School Washington University in St. Louis St. Louis, MO United States; 3 School of Journalism and Communication Shanghai University of Sport Shanghai China; 4 Division of Public Health Sciences, Department of Surgery Washington University School of Medicine in St. Louis St. Louis, MO United States; 5 Division of Data and Computational Sciences Washington University in St. Louis St. Louis, MO United States

**Keywords:** obesity, obese, overweight, voice, vocal, vocal cord, vocal cords, voice-based, machine learning, ML, artificial intelligence, AI, algorithm, algorithms, predictive model, predictive models, predictive analytics, predictive system, practical model, practical models, early warning, early detection, deep neural network, deep neural networks, DNN, artificial neural network, artificial neural networks, deep learning

## Abstract

**Background:**

The escalating global prevalence of obesity has necessitated the exploration of novel diagnostic approaches. Recent scientific inquiries have indicated potential alterations in voice characteristics associated with obesity, suggesting the feasibility of using voice as a noninvasive biomarker for obesity detection.

**Objective:**

This study aims to use deep neural networks to predict obesity status through the analysis of short audio recordings, investigating the relationship between vocal characteristics and obesity.

**Methods:**

A pilot study was conducted with 696 participants, using self-reported BMI to classify individuals into obesity and nonobesity groups. Audio recordings of participants reading a short script were transformed into spectrograms and analyzed using an adapted YOLOv8 model (Ultralytics). The model performance was evaluated using accuracy, recall, precision, and *F*_1_-scores.

**Results:**

The adapted YOLOv8 model demonstrated a global accuracy of 0.70 and a macro *F*_1_-score of 0.65. It was more effective in identifying nonobesity (*F*_1_-score of 0.77) than obesity (*F*_1_-score of 0.53). This moderate level of accuracy highlights the potential and challenges in using vocal biomarkers for obesity detection.

**Conclusions:**

While the study shows promise in the field of voice-based medical diagnostics for obesity, it faces limitations such as reliance on self-reported BMI data and a small, homogenous sample size. These factors, coupled with variability in recording quality, necessitate further research with more robust methodologies and diverse samples to enhance the validity of this novel approach. The findings lay a foundational step for future investigations in using voice as a noninvasive biomarker for obesity detection.

## Introduction

Obesity has emerged as a prominent global health concern, with its prevalence nearly tripling since 1975 and affecting a significant portion of the population worldwide [1]. This increase is especially pronounced in developing nations, partially owing to shifts in lifestyle and dietary habits [2]. Obesity serves as a precursor to various medical conditions including, but not limited to, type 2 diabetes, cardiovascular diseases, certain forms of cancer, and musculoskeletal disorders, significantly contributing to the global disease burden and elevating premature mortality rates [3]. The increased health care expenditures and reduced productivity adversely impacted the regional economy [4].

While the broad ramifications of obesity are well documented, recent scientific inquiries have begun to elucidate the potential alterations in voice characteristics that may be concurrent with obesity [5,6]. Several mechanisms are postulated to explain these alterations in vocal attributes. The deposition of adipose tissue near the vocal folds and larynx may influence vocal resonance and pitch, often resulting in variations in voice quality [7]. Restrictive lung patterns associated with obesity may lead to compromised lung volumes and capacities, subsequently impacting subglottal pressures essential for phonation [8]. Obesity induces a chronic inflammatory state, potentially altering the composition and viscosity of vocal fold tissues and affecting parameters such as jitter and shimmer [9]. In addition, the hormonal imbalances often seen in obesity can impact the elasticity and tension of laryngeal tissues, thereby influencing voice characteristics [10].

Given these insights, voice-based markers have emerged as a pioneering approach to assessing obesity [11]. The prospect of using noninvasive and readily accessible audio recordings may pave the way for advancements in diagnostic methodologies, overcoming the constraints inherent to current obesity assessment techniques [12]. This innovative method holds the potential to inform preventive health care strategies by enabling the extraction of critical health information from voice, allowing for the development of scalable, real-time, and accurate health-monitoring systems. The implications of such advancements could be especially significant in regions with limited resources, facilitating early interventions and alleviating the compounded health and economic repercussions associated with obesity. Delving into the intricate relationship between voice characteristics and obesity may enhance our understanding and propel the evolution of novel diagnostic and monitoring tools, presenting opportunities for refined strategies in obesity management and prevention.

Artificial intelligence (AI), characterized by machine and deep learning techniques, has become increasingly popular in exploring and addressing the multifaceted challenges associated with obesity [13,14]. For instance, studies have used deep neural network models to analyze face portrait photographs to predict obesity status and the risk of diabetes, showcasing the versatility and efficacy of AI in medical diagnoses and risk assessments [15]. These applications exemplify the transformative potential of AI in deriving insightful correlations and predictive analytics in the context of obesity, allowing for the development of sophisticated and nuanced approaches to studying and managing this prevalent condition.

This pilot study pioneers the exploration of using deep neural network models to predict individuals’ obesity status through analyses of short audio recordings. Participants were recorded while reading a prewritten script, and the models were developed to discern potential associations between vocal characteristics and obesity. This study constitutes the initial endeavor to explore the relationship between obesity and voice, highlighting an uncharted intersection in obesity research. Although preliminary, the study lays the groundwork in this novel domain, and relevant findings may inspire future research in voice-related health diagnostics.

## Methods

### Data

We conducted a standardized web-based survey to gather demographic information (gender and age), self-reported anthropometric measurements (height and weight), disease histories, and brief audio recordings from participants (see Multimedia Appendices 1 and 2). The participants were instructed to read a short Mandarin paragraph provided in the survey and record it using their mobile phones. Consequently, the final analysis comprised 696 participants, including 500 females and 196 males, with an average age of 24 years.

We classified study participants into 2 groups, obesity (271/696, 38.9%) and nonobesity (425/696, 61.1%), based on the standard BMI threshold of ≥28 kg/m^2^ in the Chinese population [16].

A spectrogram is a visual representation of the spectrum of frequencies in a sound signal as they vary with time, serving as an essential tool for feature extraction in audio classification tasks. Audio recordings were standardized to the WAV format and then transformed into spectrograms. The preprocessed data set was randomly partitioned into a training set of 591 audio files (591/696, 85%) and a test set of 105 files (105/696, 15%).

**Figure 1:**
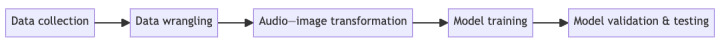
Research workflow.

Data augmentation on spectrograms involves applying various techniques such as time stretching, noise injection, and frequency masking to enhance the diversity and robustness of the data set, thereby improving the performance of machine learning models in audio classification. Data augmentation was used to balance the training set, ensuring equal representations of images labeled as obesity and nonobesity. Subsequently, a 5-fold cross-validation was performed on the balanced training set. Our workflow is illustrated in Figure 1.

### Ethical Considerations

The study was approved by the Shanghai University of Sport Ethics Committee (institutional review board #102772022RT065), with written informed consent obtained from each study participant. After negotiations, each participant received 10 yuan as compensation for participating in the study, and the data of each participant were anonymized.

### Model

We developed a neural network model to predict an individual’s obesity status using spectrogram data. Adapting the YOLO (You Only Look Once) framework [17], which is renowned for real-time object detection and image segmentation in computer vision, we fine-tuned the pretrained YOLOv8 model for our voice-based obesity classifier. To enhance model performance, we used techniques such as batch normalization, learning rate optimization, label smoothing, and early stopping. This model was constructed using Python (version 3.10.12; Python Software Foundation) and was accelerated using a Tesla V100 GPU (NVIDIA).

A comparison of the predictive performances of corresponding models applying 2 main feature extraction approaches in speech recognition was conducted. One is based on signal parameter extraction, such as Mel-frequency cepstral coefficients and Mel-filter bank features, while the other is based on spectrogram images. Table 1 delineates the performance metrics of multiple models across varied feature sets. The YOLOv8 model we applied exhibited higher performance, which is specified in italics.

**Table 1:** Overall performances of various models.

Features and model		*F*_1_-score	Sensitivity	PPV^a^	Accuracy
**Spectrogram**					
	Yolov8	*0.65^b^*	*0.69*	*0.65*	*0.70*
	CNN^c^	0.59	0.58	0.61	0.60
**MFCCs^d^+Delta-Delta**					
	CNN	0.57	0.56	0.58	0.62
	RandomForest	0.58	0.56	0.59	0.63
	MLP^e^	0.56	0.57	0.56	0.56
**MFCCs+Mel^f^**					
	CNN	0.59	0.57	0.61	0.64
	RandomForest	0.58	0.57	0.60	0.63
	MLP	0.55	0.55	0.55	0.57

^a^PPV: positive predictive value.

^b^Italics indicates higher performance.

^c^CNN: convolutional neural network.

^d^MFCC: Mel-frequency cepstral coefficient.

^e^MLP: multilayer perceptron.

^f^Mel: Mel-filter bank features.

## Results

**Figure 2:**
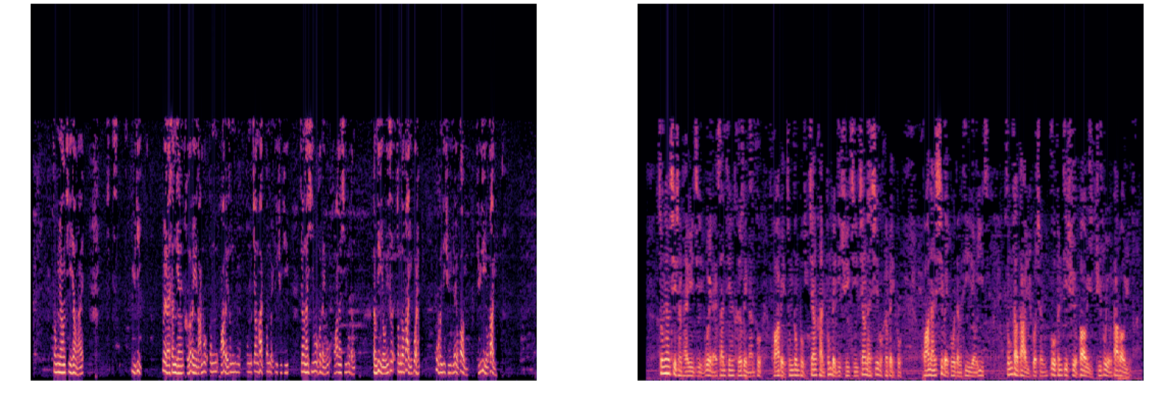
Spectrogram images labeled nonobesity (left) and obesity (right).

Figure 2 shows 2 example spectrogram images transformed from audio files labeled as nonobesity and obesity. In terms of the spectrogram, horizontal axes indicate time in milliseconds. Vertical axes indicate the frequency in hertz (Hz). Brightness indicates decibel level; the brighter it is, the higher the decibel level. The stripes in the spectrogram reflect the fundamental characteristics of a speaker's voice. Bars that are relatively parallel to the horizontal axis correspond to the formant. The distance between dark stripes perpendicular to the horizontal axis represents the period of fundamental frequency. Formant and fundamental periods are closely related to the state of the vocal tract structures.

**Figure 3:**
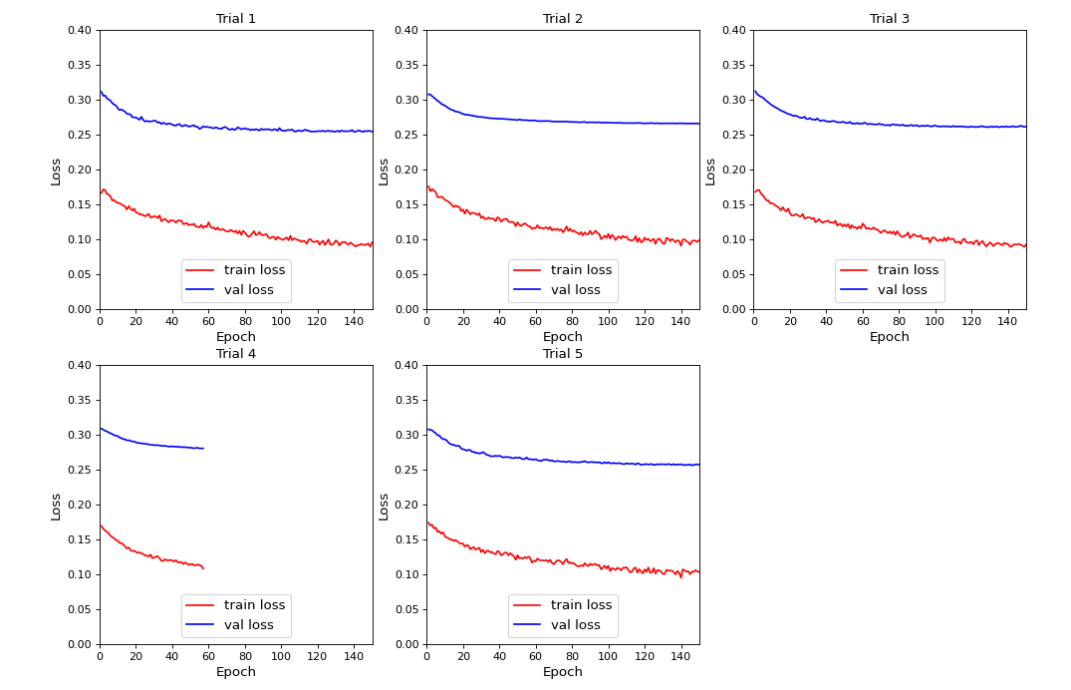
Model training using 5-fold cross-validation. Train loss: training loss; val loss: validation loss.

Figure 3 depicts the 5-fold cross-validation training process. The training loss gradually declined from around 0.15 to near zero by epoch 80. During epochs 0-80, the validation loss primarily decreased but with some fluctuations. From epochs 60-150, it began to stabilize around 0.25, with no substantial reductions afterward. The peak model performance was achieved at epoch 120, with a validation loss of 0.26 and an associated training loss of 0.10. Trail 4 displayed different epoch numbers due to a relatively small sample size and training fluctuations, which triggered the early-stop feature of the YOLOv8 model. During the training process, the curves of train loss and validation loss did not perfectly coincide at the end. However, the consistent downward and convergent trend of both indicated that the model was trained normally without overfitting or underfitting.

Table 2 reports the model performance on the test set. For the obesity category, the model yielded an *F*_1_-score of 0.53, with a recall (sensitivity) of 0.67 and a precision (positive predictive value) of 0.44. The model achieved an *F*_1_-score of 0.77 for nonobesity classifications, with a recall of 0.70 and a precision of 0.86. The overall model performance across both categories was characterized by a macro *F*_1_-score of 0.65, a recall of 0.69, a precision of 0.65, and a global accuracy of 0.70.

**Table 2:** YOLOv8 model performance on the test set.

	*F*_1_-score	Sensitivity	PPV^a^	Accuracy
Obesity	0.53	0.67	0.44	—^b^
Nonobesity	0.77	0.70	0.86	—
Overall	0.65	0.69	0.65	0.70

^a^PPV: positive predictive value.

^b^Not available.

## Discussion

This study explored the use of deep neural networks, specifically an adapted YOLOv8 model, to predict obesity status from short audio recordings. This approach aimed to identify potential relationships between vocal characteristics and obesity. Our results indicate a moderate level of accuracy in the model performance, with a global accuracy of 0.70 and a macro *F*_1_-score of 0.65. The model demonstrated a higher effectiveness in identifying nonobesity cases, as reflected by an *F*_1_-score of 0.77, compared with a lower *F*_1_-score of 0.53 for obesity classifications. These outcomes suggest that while the model shows promise, there is a need for further refinement to enhance its precision and reliability in obesity detection using vocal biomarkers.

In the context of medical diagnostics, the use of voice as a biomarker has been an emerging area of interest [18], although its application in obesity identification remains relatively unexplored. Historically, voice analysis has been successfully used in the detection of various health conditions, such as Parkinson disease, where vocal cord and speech pattern changes are indicative of the disease’s progression [19]. Similarly, in respiratory diseases, voice alterations often reflect changes in lung function and airflow [20]. The rationale behind these applications is that physiological changes, whether due to neurological, respiratory, or other systemic conditions, can manifest in measurable changes in voice characteristics [21].

The aim of our study to correlate voice characteristics with obesity aligns with this emerging trend but ventures into a relatively uncharted domain. Obesity, being a complex condition with multifactorial etiologies, may not exhibit as direct a relationship with vocal changes as seen in neurological or respiratory illnesses [22]. Nonetheless, the premise that obesity can induce physiological alterations, such as in the laryngeal tissues and respiratory system [23], provides a theoretical foundation for our exploration. The moderate success of our model in distinguishing obesity from nonobesity cases indicates a potential, albeit complex, link between obesity and voice characteristics.

The findings of this study contribute to the expanding literature on noninvasive diagnostic methods. Traditional obesity diagnosis primarily relies on physical measurements such as BMI and waist circumference, which have their limitations, including the inability to assess body fat distribution and differentiate between fat and muscle mass [24]. The prospect of supplementing these methods with voice analysis could offer a more holistic and convenient approach to obesity assessment.

Using deep neural networks, short audio recordings can predict obesity status, offering practical applications in preventive medicine, telemedicine, and public health research. It enables noninvasive early screening for obesity and related health issues such as obstructive sleep apnea [25], provides objective measures in telemedicine, and offers a cost-effective data collection approach for obesity prevalence research.

However, our study’s moderate accuracy underscores the challenges inherent in this novel diagnostic pathway. It highlights the need for further research to better understand the nuances of how obesity might specifically alter vocal characteristics and how these changes can be more accurately captured and interpreted by advanced neural network models.

This study faces several key limitations. Foremost, the reliance on self-reported BMI introduces potential inaccuracies due to measurement errors and social desirability bias [26], compromising the model’s accuracy in obesity classification. In addition, the use of a small, convenience sample limits the statistical power and generalizability of our findings, as it may not adequately represent the broader population. Variability in audio recording quality, resulting from participants using their own mobile phones, further challenges the consistency of the input data. The demographic homogeneity of the sample and the lack of consideration for other factors influencing voice characteristics, such as lifestyle choices, restrict the applicability of our findings to a wider, more diverse population. These limitations collectively underscore the need for more robust methodologies and diverse participant samples in future research to enhance the validity and applicability of voice analysis in obesity detection.

Future research should prioritize conducting a longitudinal cohort study to analyze voice changes in individuals transitioning from lean to obese phases. This will deepen our understanding of voice changes during obesity progression and enable the extraction of vocal characteristic features across different stages of obesity. Ultimately, such an approach may aid in developing causal links between obesity and vocal changes.

In sum, while our study presents an innovative approach to obesity detection and adds to the growing body of research on voice-based medical diagnostics, it also emphasizes the complexity of this endeavor and the necessity for continued research and development in this area. The potential of using voice as a noninvasive biomarker for obesity is an intriguing prospect, and our findings, though moderate in their current state, lay the groundwork for future investigations to refine and enhance this novel diagnostic method.

## Appendix

Multimedia Appendix 1 Codebook and questionnaire (in Chinese).

Multimedia Appendix 2 Codebook and questionnaire (in English).

## Abbreviations

AI: artificial intelligence
YOLO: You Only Look Once
