# Supplementary material for: Use of Deep Neural Networks to Predict Obesity With Short Audio Recordings: Development and Usability Study
Source: JMIR AI. 2024 Jul 25;3:e54885. doi: 10.2196/54885 (PMC11310637; doi:10.2196/54885)
Supplement: Multimedia Appendix 1 [file ai_v3i1e54885_app1.pdf]

Var.No.	question	variable name	variable label	code	decode
1	What is your gender?	gender	gender	0<br>1	female<br>male
2	What's your age?	age	age	/	/
3	What is your height(cm)?	height	height	/	/
4	What is your weight?(kg)	weight	weight	/	/
5	Do you have or have ever had a respiratory disease? (e.g. asthma)	respiratory_disease	respiratory disease	0<br>1	False<br>True
6	Do you have or have ever had a neurodegenerative disease? (e.g.parkinsonism)	neurodegenerative_disease	neurodegenerative disease	0<br>1	False<br>True
7	Have you ever undergone pharyngeal or laryngeal surgery?	pharynx_or_larynx_surgery	pharynx or larynx surgery	0<br>1	False<br>True
8	/	bmi_ranges	BMI ranges	0<br>1<br>2<br>3	underweight<br>healthy weight<br>overweight<br>obesity

基本信息问卷与录音实验步骤

Q1 请选择你的性别 [单选]

○ 男
○ 女

Q2 您的年龄是____周岁？ [填空]

Q3 您的身高是____cm？ [填空]

Q4 您最近一次体重测量的结果是____kg？ [填空]

Q5 您是否患有或曾经患有呼吸系统疾病（如哮喘、支气管炎等）？ [单选]

○ 是
○ 否

Q6 您是否患有或曾经患有神经系统疾病（如帕金森综合征、脑梗等）？ [单选]

○ 是
○ 否

Q7 您是否曾经接受过喉部或咽部手术？ [单选]

○ 是
○ 否

Q8 请您保持正坐姿态，按下录音键，随后持续发出“a（啊）”声并维持5秒左右 [录音]

♫

Q9 请您保持站立姿态，按下录音键，随后持续发出“a（啊）”声并维持5秒左右 [录音]

♫

Q10 请您按下录音键并以最自然舒适的状态读出以下内容：“中央气象台预计，未来三天，陕西南部、西南地区东部、华北中东部、黄淮、江汉、东北地区及江南西部和北部、华南西北部等地有一次中到大雨过程，部分地区有暴雨，局地有大暴雨。” [录音]

♫
