# Supplementary material for: Use of Deep Neural Networks to Predict Obesity With Short Audio Recordings: Development and Usability Study
Source: JMIR AI. 2024 Jul 25;3:e54885. doi: 10.2196/54885 (PMC11310637; doi:10.2196/54885)
Supplement: Multimedia Appendix 2 [file ai_v3i1e54885_app2.docx]

| Var.No. | question | variable name | variable label | code | decode |
| --- | --- | --- | --- | --- | --- |
| 1 | What is your gender? | gender | gender | 0 | female |
|  |  |  |  | 1 | male |
| 2 | What's your age? | age | age | / | / |
| 3 | What is your height(cm)? | height | height | / | / |
| 4 | What is your weight?(kg) | weight | weight | / | / |
| 5 | Do you have or have ever had a respiratory disease? (e.g. asthma) | respiratory_disease | respiratory disease | 0 | False |
|  |  |  |  | 1 | True |
| 6 | Do you have or have ever had a neurodegenerative disease? (e.g.parkinsonism) | neurodegenerative_disease | neurodegenerative disease | 0 | False |
|  |  |  |  | 1 | True |
| 7 | Have you ever undergone pharyngeal or laryngeal surgery? | pharynx_or_larynx_surgery | pharynx or larynx surgery | 0 | False |
|  |  |  |  | 1 | True |
| 8 | / | bmi_ranges | BMI ranges | 0 | underweight |
|  |  |  |  | 1 | healthy weight |
|  |  |  |  | 2 | overweight |
|  |  |  |  | 3 | obesity |

**Questionnaire and Recording Steps**

Q1 Please select your gender [Single Choice]


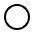
 male


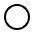
 female

Q2 How old are you? [Fill in the blank]

Q3 Your height is ____cm? [Fill in the blank]

Q4 The result of your most recent weight measurement was ____kg? [Fill in the blank]

Q5 Do you have or have ever had a respiratory disease? (e.g. asthma) [Single Choice]


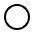
 yes


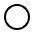
 no

Q6 Do you have or have ever had a neurodegenerative disease? (e.g.parkinsonism) [Single Choice]


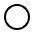
 yes


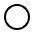
 no

Q7 Have you ever undergone pharyngeal or laryngeal surgery? [Single Choice]


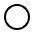
 yes


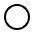
 no

Q8 Please keep sitting upright, press the record button, and then make a continuous "a (ah)" sound for about 5 seconds [recording]

| ♫ |
| --- |

Q9 Please stand up, press the record button, and then make a continuous "ah" sound for about 5 seconds [recording]

| ♫ |
| --- |

Q10 Please press the record button and read the following in the most natural and comfortable state: "The Central Meteorological Observatory predicts that in the next three days, there will be moderate to heavy rain in southern Shaanxi, eastern Southwest China, central and eastern North China, Huanghuai, Jianghan, Northeast China, western and northern Jiangnan, and northwestern South China. Some areas will have heavy rain, and some areas will have torrential rain." [Recording]

| ♫ |
| --- |
